# Remarks, Gleanings, and Extracts

**Published:** 1874-04

**Authors:** A. R. Kilpatrick

**Affiliations:** Texas


### Remarks, Gleanings and Extracts.

BY A. R. KILPATRICK, M.D., OF TEXAS.

*Coffee an Antidote for Morphia.*—Dr. J. B. Garrison, of DeWitt, Ark., narrates a very instructive and encouraging case in the *Philadelphia Med. and Surg. Reporter*, for February 7th. A white man aged 55, had by mistake, taken between 10 and 20 grs. morphia, and five hours after he took it, Dr. G. saw him. He was pulseless and almost moribund; insensible to all impressions. The Dr. considered it almost useless to try to save him, but he worked on him four hours, aided by six young men, who kept up artifical respiration; poured on cold water and used frictions vigorously; while the Doctor was constantly filling his skin, through a syringe, with solution of belladonna, having no atropia, and injected altogether 10 grs. ext. bel. and a pint of strong decoction of coffee. The coffee was thrown under the skin in every available place throughout the body. After an hour the pulse was perceptible at the wrist; at the end of the second hour the respirations were two to the minute, spasmodic and attended by rattling in the throat. Between the second and third hour he seemed to be coming round very gradually. After the fourth hour he was able to swallow and was made to drink large draughts of strong coffee, amounting in the aggregate to more than a quart. Then quinine and brandy p. r. n. catheterization was required for 48 hours. For a few weeks his memory was deficient, and he had a low form of mild fever, but finally got well; complimented the Doctor by saying, he believed he had saved his life and that the Doctor has great claim on him, but he has not settled the bill yet! Do you think he will ever pay it?

*Chloroform.*—I am pleased to see the paper in the February No. of this Journal, by Dr. J. H. Weir, of Ill., in defense of chloroform. I have been using it continuously since 1848 in every kind of case and great varieties of constitutions, and have never seen any bad effects from it. In one stout, young negro man, in 1867, who insisted on using it to have a trivial incision made for the removal of fungus or rather extra growth of ectropion in the healing of a gunshot wound involving the lids of one eye; he became slightly asphyxiated for two or three respirations, which was quickly removed by depressing the tongue and sprinkling his face with cold water. No one has died from chloroform under the treatment of any physician of my acquaintance. I know of one case dying from chloral hydrate in 1872, and another who came very near dying from it, and a young lady who was injured by æther, as she and her friends were afraid to use chloroform, while the operation for strabismus was performed by two peripatetic oculists. She inhaled æther quite an hour and her friends say, ever since that her health is impaired; how true, I do not know. All that is required in my opinion is *proper precaution.*

*The Way to Increase the Curative Power of Iodide of Potassium.*—Mr. J. T. McSweeny writes in the *British Med. Jour.*, and says: "Carbonate of ammonia greatly increases the therapeutic action of iod. potassa. The best results have attended his use of it thus combined; five grains of it with three grains of carb. ammon. is equal in strength to eight grains of the iodide alone. If there are any sores, no matter how freely they discharge, nor how offensive it may be, they soon loose the fetor and heal kindly. He has used the same combination in cases of internal aneurisms; it relieved the pain and helped to consolidate the tumors."

*Tenia Removed by Pumpkin Seed.*—I have seen two cases reported recently, where tape-worms were removed by giving pumpkin seed (pepo semina). The last is reported in the *Phila. Med. and Surg. Reporter,* February 28, by Dr. C. G. Bacon, of Fulton, New York. He first gave at 5 P. M., ℥ij. pepo sem. in a pint of milk in place of supper; at 9 P. M., same night, he gave ℥j. ol. ricini; spts. tereb., ʒij.; syrup simp., ℥ss.; also in milk. Next morning at 5 o'clock the whole worm, 18 feet long, passed out. This is the third case treated in the same successful way by Dr. Bacon in three years.

*Sewing Machines Condemned.*—Dr. Wm. Goodell, Prof. in the University of Pa., in a recent lecture, published in the *Phila. Med. and Surg. Reporter,* says: "One word here about the sewing machine. While I do not believe all that is laid to its charge, yet its treadle motion does undoubtedly lead to pelvic and portal congestions. In spite of myself I have become convinced that no woman who operates on this machine as a trade can long escape from some uterine derangement. Even its family use is not unattended with risk, because, although intermittent, it is liable to be too prolonged."

*Retention of Urine,* from atony or over-distention of the bladder, has been successfully overcome by the administration of strychnia,

*Carbolic Acid in Diabetes Mellitus.*—Drs. Ebstein and Muller, of Breslau, published in the *Berline Klin. Woch.*, of December 8, 1873, some account of their success with this remedy: They theorized that many cases of diabetes arose from the ferment which converts amyloid substances into sugar in the liver: They used carbolic acid, grs. xv.; aqua menthe, ℥ixss. Give six or seven tablespoonfuls a day, using the whole amount in three days, and repeating it if necessary.

*Child Murder.*—A negro nurse at Calvert, Texas, thrust a common dressing pin of large size, through the anterior fontanelle of a white child, which caused inflammation of the brain, convulsions and death. After death, the mother in her agony fondled the child, and running her fingers caressingly through its hair, felt the head of the pin and extracted it, which at once explained matters. The murderess was executed.

*Trichinæ* have been causing much alarm, sickness and death in Austin, San Antonio, Indianola, Dallas and some other places in Texas, during the winter. The effectual remedy or preventive, is to cook the meat thoroughly and the heat kills the worms.

*Graduates.*—The Degree of M. D. was conferred on seventy-six graduates, of the Louisville, Ky., Medical College, at its last commencement exercises. Five of these were from Texas. Nine prizes were also awarded to different graduates, for proficiency and other meritorious qualities.

*A Remedy for Headache.*—Dr. C. C. Vanderbeck, of N. J., has used with great success, equal parts of fluid ext. ergota; fluid ext. valerian, and gives thirty drops every half hour until relieved. Usually, two or three doses are sufficient.

*Intussusception* has been frequently overcome by injecting into the bowels, first a solution of bicarb soda, followed with tartaric acid.

*Neurosis and Neuralgia* can be relieved by the administration of valerianic ether. It is more palatable than other forms of valerian and not liable to produce nausea.

*Syphilitic Neuralgia* is treated successfully by Dr. Hammond, of New York, with bromide of calcium. Dissolve one ounce in four ounces of water, and give a tablesponful morning and noon, and two tablespoonfuls at bed-time.

*Gonorrhœa* has been cured, after the acute symptoms are passed, by injecting *ter in die* a syringe full of a solution of 15 to 22 grains chloral, in 3⅛ ounces water.

